# The When, What & How of Measuring Vitamin D Metabolism in Clinical Medicine

**DOI:** 10.3390/nu10040482

**Published:** 2018-04-13

**Authors:** Niek F. Dirks, Mariëtte T. Ackermans, Paul Lips, Renate T. de Jongh, Marc G. Vervloet, Robert de Jonge, Annemieke C. Heijboer

**Affiliations:** 1 Endocrine Laboratory, Department of Clinical Chemistry, VU University Medical Center, 1007 MB Amsterdam, The Netherlands; n.dirks@vumc.nl (N.F.D.); r.dejonge1@vumc.nl (R.d.J.); 2 Laboratory of Endocrinology, Department of Clinical Chemistry, Academic Medical Center, 1100 DDAmsterdam Zuidoost, The Netherlands; m.t.ackermans@amc.uva.nl; 3 Department of Internal Medicine and Endocrinology, VU University Medical Center, Amsterdam Movement Sciences, 1007 MB Amsterdam, The Netherlands; p.lips@vumc.nl (P.L.); rt.dejongh@vumc.nl (R.T.d.J.); 4 Department of Nephrology, VU University Medical Center, 1007 MB Amsterdam, The Netherlands; m.vervloet@vumc.nl

**Keywords:** vitamin D, metabolism, 25-hydroxyvitamin D, 1,25-dihydroxyvitamin D, 24,25-dihydroxyvitamin D, LC-MS/MS, immunoassay

## Abstract

We now have the ability to measure a number of different vitamin D metabolites with very accurate methods. The most abundant vitamin D metabolite, 25-hydroxyvitamin D, is currently the best marker for overall vitamin D status and is therefore most commonly measured in clinical medicine. The added value of measuring metabolites beyond 25-hydroxyvitamin D, like 1,25-, and 24,25-dihydroxyvitamin D is not broadly appreciated. Yet, in some more complicated cases, these metabolites may provide just the information needed for a legitimate diagnosis. The problem at present, is knowing when to measure, what to measure and how to measure. For 25-hydroxyvitamin D, the most frequently used automated immunoassays do not meet the requirements of today’s standards for certain patient groups and liquid chromatography-tandem mass spectrometry is the desired method of choice in these individuals. The less frequently measured 1,25-dihydroxyvitamin D metabolite enables us to identify a number of conditions, including 1α-hydroxylase deficiency, hereditary vitamin D-resistant rickets and a number of granulomatous diseases or lymphoproliferative diseases accompanied by hypercalcaemia. Furthermore, it discriminates between the FGF23-mediated and non-FGF23-mediated hypophosphatemic syndromes. The 24,25-dihydroxyvitamin D metabolite has proven its value in the diagnosis of idiopathic infantile hypercalcaemia and has the potential of having value in identifying other diseases. For both metabolites, the understanding of the origin of differences between assays is limited and requires further attention. Nonetheless, in every way, appropriate measurement of vitamin D metabolism in the clinical laboratory hinges eminently on the comprehension of the value of the different metabolites, and the importance of the choice of method.

## 1. Introduction

With the ever-growing family of measurable vitamin D metabolites and techniques to assess them, comes a predicament. How does a clinician decide when measurement of vitamin D metabolism benefits the diagnostic process? What metabolic product of vitamin D provides the necessary answers, and how best to measure it? The goal of this review is to clarify the value of measuring vitamin D metabolites in diagnostics and to illustrate which metabolite is to be measured under which circumstances, and why. Additionally, the choice of method is discussed, as this is an important aspect of appropriate vitamin D metabolism measurement. 

The paper is structured as follows. Section 2 is dedicated to the metabolism of vitamin D. Knowledge of the processes and the stimulatory and inhibitory factors involved in this ingenious metabolic pathway is a prerequisite for understanding the benefits and relevance of measuring the different metabolites. Section 3 focuses on the when and what, situations where the major vitamin D metabolites may contribute to a fitting diagnosis or monitoring of a disease. Section 4, on the how, explores the different methods a present-day laboratory may offer to measure the desired metabolites. As not all available methods are suitable for every situation, knowing the flaws and pitfalls of the various measurement procedures is important to prevent a misdiagnosis or missed diagnosis. In the end, everyone benefits from a faster, more reliable and more economical way to achieve proper diagnosis by knowing when to measure, what to measure and how to measure vitamin D metabolites in a clinical setting.

## 2. Vitamin D Metabolism

**Figure 1:**
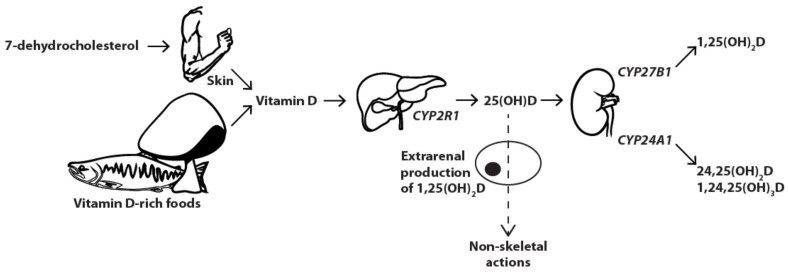
Vitamin D metabolism. From the production of vitamin D in the human skin or ingestion from certain vitamin D-rich foods to the final metabolisation into active (1,25(OH)_2_D) and largely inactive metabolites (24,25(OH)_2_D and 1,24,25(OH)_3_D). 25(OH)D: 25-hydroxyvitamin D; 1,25(OH)_2_D: 1,25-dihydroxyvitamin D; 24,25(OH)_2_D: 24,25-dihydroxyvitamin D; 1,24,25(OH)_3_D: 1,24,25-trihydroxyvitamin D.

Vitamin D metabolism (Figure 1) is a complex and ingenious process proceeding inside multiple organs within the human body to maintain calcium homeostasis and control bone metabolism. It all starts with 7-dehydrocholesterol which, when exposed to UVB light in the human skin, transforms into vitamin D_3_ through intermediate isomer pre-vitamin D_3_. Alternatively, a small portion of vitamin D_3_ may be directly ingested by the consumption of animal products. Another isomeric form, vitamin D_2_, is not synthesised by our own body, but may be extracted from some plants and fungi. Both forms behave similarly and undergo the same metabolic process. Both activated forms also bind to the vitamin D receptor (VDR), although the active metabolite of vitamin D_3_ has proven to be more potent [1]. From here, if there is no need for the distinction between the two isomers, vitamin D and associated metabolic products refer to both species. Cutaneously synthesised or ingested vitamin D is subsequently transported to the liver, usually bound to a vitamin D binding protein (DBP), where hydroxylation on the 25-position by hepatic CYP2R1 (25-hydroxylase) yields 25-hydroxyvitamin D (25(OH)D). The hormonally active 1,25-dihydroxyvitamin D (1,25(OH)_2_D) is produced in the proximal renal tubule by the mitochondrial CYP27B1 enzyme (1α-hydroxylase), after a second hydroxylation at the 1-position. To retain 1,25(OH)_2_D concentrations within the strict boundaries required for appropriate calcium homeostasis and bone metabolism, both 1,25(OH)_2_D and 25(OH)D may undergo further hydroxylation by renal CYP24A1 (24-hydroxylase), leading, respectively, to 1,24,25-trihydroxyvitamin D and 24(R),25-dihydroxyvitamin D (24,25(OH)_2_D), with negligible affinity for the VDR. In addition, all of the aforementioned metabolites are subject to C-3 epimerisation resulting in products with lower VDR and DBP binding affinities and reduced activity compared to their unepimerised counterparts [2].

This metabolic process is tightly controlled by a number of regulators. Parathyroid hormone (PTH) stimulates production of 1,25(OH)_2_D by upregulating CYP27B1 gene expression, while inhibiting expression of CYP24A1. Fibroblast growth factor 23 (FGF23) inhibits CYP27B1 and stimulates CYP24A1, thus preventing the production of 1,25(OH)_2_D and stimulating its catabolism [3]. Calcium decreases CYP27B1 activity directly and through inhibition of the action as exerted by PTH, all leading to lower 1,25(OH)_2_D concentrations. Lack of phosphate (hypophosphatemia) results in the opposite, by stimulating CYP27B1 and inhibiting CYP24A1 expression. 1,25(OH)_2_D itself supresses CYP27B1 and stimulates CYP24A1, thereby promoting its own degradation when excessively present. Apart from the well-documented renal conversion of 25(OH)D to 1,25(OH)_2_D for endocrine purposes under the regulatory control of PTH, FGF23 and 1,25(OH)_2_D, other vitamin D activating sites exist. In fact, many tissues harbour cells capable of metabolising 25(OH)D and additionally express the VDR, resulting in the possibility of intracrine, autocrine and paracrine effects [4]. These include immune cells, different types of epithelial cells, bone cells and parathyroid cells. Control of 1,25(OH)_2_D production in these extrarenal cells differs from regulation within the kidneys, as receptors for PTH are lacking. Here, the most important limiting factor is the availability of substrate [5]. The non-classical actions of 1,25(OH)_2_D in these cells involve local regulation of PTH secretion in the parathyroid gland, local regulation of insulin secretion in the pancreatic beta cells, modulating immune cell response in activated inflammatory cells and regulation of proliferation and differentiation in the keratinocytes [4].

Regulation of 25(OH)D metabolism, mediated by the two cytochrome P450 enzymes, is of crucial importance. When out of balance, serious complications may occur. Too high 1,25(OH)_2_D concentrations result in increased calcium absorption from the intestines leading to hypercalcaemia and associated serious effects. Too low concentrations will mean a shortage of free calcium or hypocalcaemia and the accompanying symptoms. As such, 1,25(OH)_2_D, is almost perpetually within normal limits, even when, based on 25(OH)D concentrations, the patient displays vitamin D deficiency.

## 3. When & What: Assessing Vitamin D Metabolism Misbalance

### 3.1. 25(OH)D

Serum total 25(OH)D, the summation of 25(OH)D_2_ and 25(OH)D_3_, is the best reflection of vitamin D status. It is a better marker for vitamin D status than circulating 1,25(OH)_2_D, as the latter is tightly regulated and its levels are strictly kept between limits even when adverse effects start to occur. Next to renal 25(OH)D metabolism, the local extrarenal conversion of 25(OH)D into 1,25(OH)_2_D, accounts for the hormone’s non-calcaemic effects. Notably, these extrarenally produced 1,25(OH)_2_D levels will not be mirrored by the concentration of 1,25(OH)_2_D in the systemic circulation if the locally produced 1,25(OH)_2_D does not leave the extracellular confinements to enter the bloodstream. In contrast, circulating 25(OH)D concentrations reflect the cellular 25(OH)D concentrations prior to conversion and as such, represent the available substrate for extrarenal 1,25(OH)D synthesis.

Advantageously, as 25(OH)D is the main circulating metabolite of vitamin D, high concentration facilitates its measurement.

Experts in the field of vitamin D have provided us with several reviews on the measurement of vitamin D status and interpretation of the results [6,7,8,9]. Nevertheless, disagreement still prevails, as consensus on the precise 25(OH)D concentrations representing vitamin D deficiency, insufficiency, sufficiency and intoxication has not yet been reached. One could argue that the 25(OH)D concentrations found in representative groups of individuals, asymptomatic for disease, are to be considered normal and desired [10,11,12]. However, many experts believe, as a result of changing diets and reduced levels of sun exposure, a large part of the world population is not vitamin D sufficient, and should therefore be supplemented [7,8,9,13,14]. They are convinced these higher 25(OH)D concentrations are necessary to prevent morbidity. According to the Institute of Medicine, serum 25(OH)D levels of 50 nmol/L (20 ng/mL) are sufficient to ensure skeletal health and only levels below 30 nmol/L (12 ng/mL) are to be considered universally inadequate, while levels between 30 and 50 nmol/L (12–20 ng/mL) potentially are, depending on the individual [15]. The Endocrine Society defines deficiency as 25(OH)D levels below 50 nmol/L (20 ng/mL) but recommends at least levels above 75 nmol/L (30 ng/mL), and preferably between 100 and 150 nmol/L (40–60 ng/mL), achieved by daily supplementation. However, both institutes deem population wide screening for vitamin D deficiency unnecessary and advise only to screen populations at risk [16]. Such groups include obese individuals, pregnant and lactating women, individuals with darker skin pigmentation, older adults with a history of falls or fractures, patients with rickets, osteomalacia, osteoporosis, chronic kidney disease (CKD), liver failure, hyperparathyroidism, granuloma-forming disorders, some lymphomas, malabsorption issues or nephrotic syndrome and patients on a wide range of medications, including anticonvulsants, glucocorticoids, antifungals and medication to treat HIV/AIDS [15,16].

### 3.2. 1,25(OH)_2_D

Counterintuitively, measuring 1,25(OH)_2_D as a marker for vitamin D homeostasis is irrelevant in most cases, as it will be within reference range, even when one is considered vitamin D deficient and experiences associated adverse effects. Specific conditions, however, may require the assessment of 1,25(OH)_2_D, of which concentrations normally range between 59 and 159 pmol/L (25–66 pg/mL) [17]. Several diseases may either increase or decrease 1,25(OH)_2_D concentration to undesirable levels [6]. These are all characterised by disturbed vitamin D metabolism on the level of 1,25(OH)_2_D production, which is often not reflected by altered 25(OH)D concentrations. The disorders may be classified according to three distinct origins of the 1,25(OH)_2_D production imbalance. First, and most common are disorders of the 1,25(OH)_2_D producing CYP27B1 enzyme. A second arises from mutations in the vitamin D receptor (VDR), rendering it unresponsive or less responsive to its substrate. Finally, a third category consists of conditions characterised by excessive extrarenal conversion of 25(OH)D into 1,25(OH)_2_D. 1α-hydroxylase deficiency, also known as vitamin D-dependent rickets type 1 or pseudo-vitamin D deficiency rickets, is an autosomal recessive disorder exemplary for the first category. This rare disease is caused by an inactivating mutation in CYP27B1, resulting in abnormally low 1,25(OH)_2_D concentrations and early onset of rickets [18]. A number of disorders presenting as hypophosphatemic syndromes also belong to the first category. Many, but not all, of these syndromes are mediated by FGF23, and 1,25(OH)_2_D is an excellent marker to differentiate between the FGF23-mediated and non-FGF23-mediated syndromes. The genetic disorders X-linked hypophosphatemia (XLH), autosomal dominant hypophosphatemic rickets (ADHR), and autosomal recessive hypophosphatemic rickets 1,2 and 3 (ARHR1, ARHR2 and ARHR3), are all examples of the FGF23-mediated kind, as is the acquired tumour induced osteomalacia (TIO) [19,20,21,22,23,24,25,26]. Additionally, other rare disorders may manifest as FGF23-mediated hypophosphatemia, including osteoglophonic dysplasia, McCune–Albright syndrome, epidermal nevus syndrome, neurofibromatosis, hypophosphatemic rickets with hyperparathyroidism and Jansen metaphyseal chondrodysplasia [27,28,29,30,31,32]. Elevated FGF23 levels result in inhibition of CYP27B1 and subsequent low or inappropriately normal 1,25(OH)_2_D concentrations [33]. The non-FGF23-mediated disorders displaying hypophosphatemia, including hereditary hypophosphatemic rickets with hypercalciuria, idiopathic hypercalciuria and Fanconi syndrome display similar phenotypes yet, in contrast to the FGF23-mediated syndromes, result in normal FGF23 levels and normal or appropriately elevated 1,25(OH)_2_D concentrations [34,35,36]. Hereditary vitamin D-resistant rickets, also known as vitamin D-dependent rickets type 2 belongs to the second category and is caused by a mutation in VDR, rendering it unresponsive to its substrate, resulting in hypocalcaemia and early onset rickets. Very high 1,25(OH)_2_D concentrations are found in these individuals. Examples of the third category, sarcoidosis, tuberculosis, rheumatoid arthritis, inflammatory bowel disease and the lymphoproliferative disorders, are all characterised by the formation of lumps of inflammatory cells, or granulomas, with the capability of hydroxylation of 25(OH)D to form 1,25(OH)_2_D, facilitating antimicrobial and anti-inflammatory immune responses. This extrarenal 1α-hydroxylation by local CYP27B1 is not controlled by PTH, FGFG23, phosphate or 1,25(OH)_2_D, but is regulated by local factors such as IFN-γ and IL15 and dependent on the availability of substrate [37]. When excessive, the locally produced 1,25(OH)_2_D may escape the confinements of the intracellular space, spill over to the systemic circulation, and raise blood 1,25(OH)_2_D concentrations to abnormally high levels [38]. Importantly, extrarenal 1α-hydroxylation is often associated with low bone mineral density. 1,25(OH)_2_D assessment is therefore not only of great clinical value in the diagnosis of the disease but also in the prevention of complications in the form of bone disorders [39,40].

In summary, requesting measurement of 1,25(OH)_2_D could be of use on suspicion of 1α-hydroxylase deficiency where very low 1,25(OH)_2_D concentrations are found. 1,25(OH)_2_D measurement also aids in separating the disorders presenting as hypophosphatemic syndromes mediated by FGF23, where normal to low 1,25(OH)_2_D concentrations are found, from the non-FGF23-mediated disorders with normal to high 1,25(OH)_2_D concentrations. Very high 1,25(OH)_2_D concentrations indicate hereditary vitamin D-resistant rickets or the presence of excessive extrarenal 1α-hydroxylation by granulomatous or lymphoproliferative diseases.

### 3.3. 24,25(OH)_2_D

The CYP24A1 enzyme catabolises excess 25(OH)D by converting it to the inactive 24,25(OH)_2_D. Its expression is not limited to the kidneys. Similar to CYP27B1, it expands to many different cell types, allowing for the local regulation of 25(OH)D and 1,25(OH)_2_D in target cells [41]. In a group of 92 healthy individuals evenly covering all age decades between 20 and 70 years, the 95% confidence interval of measured 24,25(OH)_2_D concentrations ranged from 0.5 to 11 nmol/L (0.2–4.6 ng/mL) and the 25(OH)D/24,25(OH)_2_D ratio ranged from eight to 27. Concentrations of 24,25(OH)_2_D appeared to be independent from the presence or absence of vitamin D deficiency (own data).

Two articles by St Arnaud in the late nineties elaborately postulated 24,25(OH)_2_D as an additional active metabolite of 25(OH)D. A role in cartilage development, embryogenesis and in regulating bone growth, development and repair was proposed [42,43]. How 24,25(OH)_2_D exerted these effects remained unclear, which led the authors to theorise on the existence of a specific nuclear or membrane receptor for 24,25(OH)_2_D. To this day, researchers are still questioning the active physiological role of 24,25(OH)_2_D and the possible existence of a dedicated receptor. Nonetheless, irrespective of its supposed activity, measuring 24,25(OH)_2_D undeniably has value, as several studies have proven during the years following St Arnaud’s articles. Unsurprisingly, the 25(OH)D/24,25(OH)_2_D ratio is an indicator of CYP24A1 activity and vitamin D catabolism therewith [44,45]. In that capacity, the ratio can identify a rare genetic disorder, idiopathic infantile hypercalcaemia (IIH). Infants suffering from this disorder display severe hypercalcaemia and suppressed PTH levels due to their severely impaired capacity to catabolise 25(OH)D and 1,25(OH)_2_D, as a result of an inactivating mutation in the gene coding for CYP24A1 [45,46]. Similarly, less harsh inactivating mutations may result in nephrocalcinosis and nephrolithiasis in adult life, secondary to hypercalciuria and often hypercalcaemia [47,48,49]. The effectiveness of the 25(OH)D/24,25(OH)_2_D ratio was further established in a cohort of hypercalcaemic patients, in which it correctly identified those patients harbouring an inactivating CYP24A1 mutation [50]. Aditionally, an elevated 25(OH)D/24,25(OH)_2_D ratio predicts vitamin D defiency to at least a similar extent as increased PTH does, and might be of potential use as an indicator of vitamin D deficiency [45,51].

In conclusion, the 25(OH)D/24,25(OH)_2_D ratio is an unambiguous marker for vitamin D catabolism and may identify patients with hypercalcaemia secondary to CYP24A1 mutations, such as patients with IIH.

### 3.4. Other Metabolites

Beyond 25(OH)D, 1,25(OH)_2_D and 24,25(OH)_2_D many more vitamin D metabolites have been identified, yet these are scarcely measured. With the current state of techniques, many of the metabolites circulating at concentrations below that of 1,25(OH)_2_D will be unquantifiable and the value of these metabolites for diagnostics remains elusive. Nevertheless, future studies may find ways of detecting and using these metabolites to provide new diagnostic tools in the years to come.

## 4. How: Importance of the Choice of Method

### 4.1. 25(OH)D

25(OH)D can be measured in many different ways, yet two techniques dominate: automated immunoassay and liquid chromatography coupled to tandem-mass spectrometry (LC-MS/MS). According to the Vitamin D External Quality Assessment Scheme (DEQAS), overseeing method comparability across 54 countries, in October 2017, ~76% of the 871 participating laboratories used an automated immunoassay to measure their 25(OH)D, while ~18% used LC-MS/MS, ~3% used a manual immunoassay and ~2% used HPLC. During recent years, several studies addressed the limitations of the automated, and less frequently the manual immunoassays, when measuring in patients [52,53,54,55,56,57]. While in healthy individuals, the assays correlate nicely with the standardised LC-MS/MS method, measuring 25(OH)D in patient groups has revealed huge deviations [52,55,56,58]. This brings to light some of the major issues with the automated and manual immunoassays. First, the lack of sample preparation in the automated immunoassays allows for the emergence of severe interferences from miscellaneous origins. Differing concentrations of DBP, which are greatly affected in certain populations, such as in pregnant women, intensive care patients and patients with liver failure, may be a common problem affecting results [55,58,59]. Similarly, the automated immunoassays often have incomprehensible difficulties measuring 25(OH)D in haemodialysis patients and osteoporotic patients [52,55,57]. Unlike with the automated immunoassays, sample preparation for the HPLC, manual immunoassays and LC-MS/MS methods is adaptable and can be optimised to reduce any adverse effects that varying DBP concentrations or other contributing factors may bring. A second origin of bias for both the automated and manual immunoassays is related to the specificity of the used antibodies. As mentioned before, 25(OH)D may be present in one of two forms, 25(OH)D_2_ or 25(OH)D_3_. Immunoassays are generally not able to distinguish the two, and report these together as total 25(OH)D. As the antibodies in the immunoassay do not bind 25(OH)D_2_ to a similar extent as they bind 25(OH)D_3,_ this leads to the under- or over-estimation of total 25(OH)D [60,61,62,63,64]. Alarmingly, many automated immunoassay manufacturers report considerably different cross-reactivity percentages for 25(OH)D_2_ compared to the observed values by independent researchers [65,66]. This is particularly problematic in countries were supplementing with vitamin D_2_ is more common, for example the USA. On the contrary, LC-MS/MS has no problem measuring 25(OH)D_2_ alongside 25(OH)D_3_, as the mass difference of 12 Da is easily separated by mass spectrometry. Another inherent specificity problem for the assays using antibodies, is cross-reactivity with other vitamin D metabolites. 24,25(OH)_2_D_3_, present in about 10–15% of the 25(OH)D concentration, has proven to greatly impact immunoassays and produces falsely high results [51,67,68]. Similar to 25(OH)D_2_, 24,25(OH)_2_D_3_, has a different mass to 25(OH)D_3_ and poses no threat to the reliability of LC-MS/MS methods [67]. Epi-25(OH)D, the result of epimerisation of 25(OH)D, with levels that are highest in neonates up to one year of age and present in most adults at about 9% of 25(OH)D, does not influence the results of immunoassays, as antibodies do not recognise it [61,69,70]. Many LC-MS/MS assays, on the other hand, experience difficulties distinguishing the two epimers due to their equal mass and their similar affinity for most LC columns, resulting in an overestimation, and potential misclassification, of total 25(OH)D [61,69,71,72]. Several LC-MS/MS assays now use non-C18 columns enabling them to chromatographically separate the epimer and measure it independently of 25(OH)D, despite the identical masses [45,68,73,74,75,76]. 

When the first LC-MS/MS methods for 25(OH)D made their entry, DEQAS reported unflatteringly high interlaboratory differences in the measurement of 25(OH)D. Carter and Jones showed these differences were often attributable to the use of in-house calibrated standards, and significantly abated after the use of a shared standard [77]. Indeed, another study showed LC-MS/MS methods for 25(OH)D generally correlated nicely and small biases originated from differences in calibration procedures [78]. Thanks to the Vitamin D Standardization Program (VDSP) and the target value DEQAS now provides for participants, laboratories operating miscalibrated LC-MS/MS methods have the possibility to standardise their methods using the reference measurement procedure, which is crucial for their performance.

In earlier years, automated immunoassays had largely replaced manual assays with their easy operation and higher throughput. However, all the aforementioned flaws of the immunoassays that were brought to light in certain patient groups, have now tipped the scales in favour of the LC-MS/MS. Today, LC-MS/MS is slowly replacing automated immunoassays in clinical laboratories. Due to the necessity of more complex and expensive equipment and highly skilled technicians, most of the smaller laboratories still depend on the manual and automated immunoassays. Manual immunoassays may still be well-suited for 25(OH)D measurement in patient groups where adequate sample preparation resolves the specificity issues of the technique. Automated immunoassays may still be suitable for the determination of 25(OH)D concentrations in large cohorts of healthy individuals, where interferences and supplementation are less frequent. We must conclude however, that measuring 25(OH)D in a clinical setting benefits greatly from the transition from automated immunoassays to LC-MS/MS. Still, attention to the specifications and quality of LC-MS/MS methods is advised. Particular specifications, such as separately quantifying epi-25(OH)D_3_ in neonates, may be crucial. Standardisation and the resulting good agreement with the DEQAS target value or performance within the VDSP defines high quality methods. Without data on method agreement, DEQAS performance or standardisation, the quality and suitability of a method is indefinable and the possibility of misclassification exists.

Of note, a few laboratories still run HPLC methods for their 25(OH)D determination. While most of the advantages of LC-MS/MS over immunoassays also hold true for HPLC, mass spectrometers have largely replaced the UV detectors used in combination with HPLC, for their superior sensitivity and specificity. The advantages and disadvantages of today’s two most widely used techniques, immunoassays and LC-MS/MS for 25(OH)D measurement are summarised in Table 1.

All things considered, LC-MS/MS is the preferred technique for 25(OH)D measurement in patients, without the knowledge of any present interferences that might hinder its determination. The use of immunoassays is not advised in specific patient groups, including IC, haemodialysis, osteoporotic and liver failure patients, pregnant women, neonates and individuals on D_2_ supplementation, and caution is warranted in unstudied patient groups.

### 4.2. 1,25(OH)_2_D

As a result of the very low concentrations of circulating 1,25(OH)_2_D and the specialised circumstances under which its analysis is deemed necessary, fewer laboratories conduct this measurement. Of the 165 methods that participated in the DEQAS distribution of October 2017, ~75% of laboratories used an automated immunoassay to measure 1,25(OH)_2_D, ~17% used a manual immunoassay and ~9% used LC-MS/MS. Similar to measuring 25(OH)D, the methods for measuring 1,25(OH)_2_D each have their advantages and disadvantages (Table 1), making the selection of one of them difficult and relevant. Previously, the field of 1,25(OH)_2_D measurement was dominated by radio-immunoassays. These assays often suffered from substantial cross-reactivity with other vitamin D metabolites and were unable to produce evincive results [79,80]. In the case of the 1,25(OH)_2_D assays, the newest automated immunoassays perform similarly to LC-MS/MS and no apparent specificity problem exists. Here, the overall unaccountable lack of good correlations between assays on all platforms is most problematic [17,81,82]. Without the presence of a reference method, identifying the methods that deviate is very difficult, if not impossible. A recently developed automated immunoassay shows good correlations to some of the LC-MS/MS methods that include an immunopurification step in their sample preparation, while lesser correlations are found with others that do not [82,83,84]. LC-MS/MS methods without immunopurification included in their sample preparation might suffer from isobaric interferences, such as 1β-25-dihydroxyvitamin D_3_, which would not be co-captured by the antibodies in the automated immunoassay [85]. Additionally, as with 25(OH)D, differences in calibration procedures are a likely cause of deviations and methods would substantially benefit from a target value provided by DEQAS and subsequent standardisation.

While immunoassays are quite easily capable of measuring very low concentrations of 1,25(OH)_2_D due to the inherent amplifying nature of the technique, LC-MS/MS assays struggle gaining enough sensitivity to accurately measure in the lower ranges of physiological concentrations. One way to overcome this is the use of more sophisticated chromatography. By making use of a 2D chromatography system, background noise is reduced and befouling of the MS instrument diminished. It comprises of two parallel connected columns. Only when the analytes of interest elute from the first column is it connected to the second column. Before and after this moment, flow is directed towards a waste. Another option is derivatisation of the vitamin D molecule. By chemical transformation of the molecule into something more easily ionised, sensitivity is improved. For the vitamin D molecules, several derivatisation agents are available. Most of them are based on the same Diels–Alder reaction taking place on the diene structure adjacent to the A-ring in the backbone of vitamin D. The diophenile molecules differ in the groups attached to their triazole backbone. The two most widely used derivatisation agents are PTAD and Amplifex.

As expected, the assays using a derivatisation agent generally report lower limits of quantification (LOQs), with levels up to 2.5 pmol/L [17,75,80,86,87,88]. The assays not using derivatisation require more elaborative sample preparation (immunopurification), increased sample volume (>0.5 mL), more sophisticated LC-MS/MS systems (µLC or 2D chromatography) or do not reach desirable LOQs [74,88,89,90,91,92]. The lowest LOQs have been reached using immunopurification followed by PTAD derivatisation [17,80]. Using Amplifex has the advantage of not requiring an immunopurification step, which makes it possible to include additional vitamin D metabolites aside from 1,25(OH)D_3_ and 1,25(OH)D_2_, but impedes the chromatographic separation of epimers. 

It is difficult to convincingly argue in favour of one technique over the other in the case of 1,25(OH)_2_D. When separate quantification of 1,25(OH)_2_D_3_ and 1,25(OH)_2_D_2_ is preferable, in countries with vitamin D_2_ supplementation, LC-MS/MS is the only option. Notwithstanding, in the case of 25(OH)D, standardisation and subsequent amelioration of LC-MS/MS methods exposed the pitfalls of the immunoassays. With the 1,25(OH)_2_D methods, a similar chain of insights may lay ahead. Without the availability of a reference method, though, standardisation is not an option and identifying the origin of biases between methods and prescribing necessities for 1,25(OH)_2_D measurements in clinical diagnostics is difficult. Nonetheless, three things are evident from the available data. First, there is no incontestable difference of outcome between the newest automated immunoassays and LC-MS/MS. Second, when using a dedicated LC-MS/MS method for 1,25(OH)_2_D, incorporation of immunopurification in the sample preparation generally leads to a method with greater sensitivity and specificity. Third, when measuring multiple vitamin D metabolites at the same time, immunopurification is not applicable and LC conditions and sensitivity optimisation need considerable attention to accurately measure each metabolite at endogenous concentrations.

### 4.3. 24,25(OH)_2_D

Several laboratories have developed methods for the quantification of 24,25(OH)_2_D. Those participating in the DEQAS distribution all use LC-MS/MS, despite the existence of a 24,25(OH)_2_D radio-immunoassay. The advantages and disadvantages of LC-MS/MS methods are depicted in Table 1. Most of them, but not all, use an isotopically labelled standard to aid them in accurate measurement. The methods measuring 24,25(OH)_2_D have usually incorporated additional vitamin D metabolites in their analysis. One thing to consider is the application of a derivatisation agent. 24,25(OH)_2_D is present at about 10% of the 25(OH)D concentration. As a result, similar to 1,25(OH)_2_D, it is not easily measured by our current high-end LC-MS/MS systems without extra efforts to improve sensitivity. One of the options is derivatising the metabolite with a derivatisation agent, like PTAD, DMEQ-TAD or Amplifex [45,75,88,93]. This increases sensitivity considerably, yet unfortunately PTAD thwarts the separate quantification of epi-25(OH)D_3_. Without derivatisation, higher LOQs are reported, additional efforts in other areas have to be undertaken to acquire similar LOQs, or higher sample volumes are required [44,68,73,74,86,94,95].

A candidate reference method was published by Tai and Nelson with high precision and accuracy [96]. Using five serum samples identified by the NIST as Standard Reference Material (SRM) and 30 samples from the DEQAS distribution, the candidate reference method and five laboratories routinely measuring 24,25(OH)_2_D assessed their comparability [97]. Mean biases of the participants ranged from −15% to 36% for the SRM samples and from 6% to 15% for the DEQAS samples. A significant SD of the mean bias for some of the laboratories indicated room for precision improvement. Overall, significant biases with the candidate reference method for 24,25(OH)_2_D shows we are long from uniform measurement of the metabolite. This is especially problematic when measuring the 25(OH)D/24,25(OH)_2_D ratio as it becomes unreliable and inaccurate when either one of the two methods is unstandardised. Further efforts for standardisation, including the availability of the SRMs and provision of a target value by DEQAS, are a necessity for uniform measurement and interpretation.

The measurement of 24,25(OH)_2_D remains mainly research driven instead of clinical, and data on method comparison is scarce. In consequence, designating high quality methods is difficult. In the coming years, 24,25(OH)_2_D will probably be more widely incorporated in clinical laboratories and attention to standardisation will lead to its solid anchoring within vitamin D metabolism diagnostics.

### 4.4. Other Metabolites

A number of the aforementioned LC-MS/MS methods measure additional metabolites. Where, in the case of epi-25(OH)D_3_, the value of measurement in neonates is well established, for other metabolites, such as 4β,25(OH)_2_D_3_, 23(R),25(OH)_2_D_3_ and 24(OH)D, the significance is not clear. These metabolites are usually incorporated in LC-MS/MS methods after being identified as an interference, and subsequently chromatographically separated and quantified. To date, a real reason to quantitatively measure these additional vitamin D metabolites is lacking, and nothing can be stated about the importance of the choice of method.

## 5. Discussion and Conclusions

While the reasons for measuring 25(OH)D have often been described, the value of measuring its metabolites, 1,25(OH)_2_D and especially 24,25(OH)_2_D, remains largely unappreciated. In short, indications where measurement of 1,25(OH)_2_D may contribute to diagnosis or aid in monitoring treatment include conditions where production of 1,25(OH)_2_D is heavily disturbed, resulting in either a shortage of 1,25(OH)_2_D, such as in 1α-hydroxylase deficiency, or an overabundance of 1,25(OH)_2_D, such as in hereditary vitamin D-resistant rickets, sarcoidosis, tuberculosis, rheumatoid arthritis, inflammatory bowel disease and lymphoproliferative diseases. 1,25(OH)_2_D also helps to identify the hypophosphatemic syndromes mediated by FGF23, including XLH, ADHR, ARHR and TIO. Measuring 24,25(OH)_2_D enables identification of CYP24A1 mutations leading to impaired catabolism of the hormone and associated hypercalcaemia, such as in IIH.

For determination of these analytes in the clinical laboratory, we have long relied on immunoassays. The automation of assays increased throughput and reduced complexity. However, the emergence of LC-MS/MS has provided us with new insight into their limitations. Yes, automated immunoassays may be fast, less laborious and sensitive, but the superiority of LC-MS/MS assays regarding specificity is evident and has revealed some serious issues that hinder immunoassays from producing reliable results in certain patient groups. The problems that may arise when selecting a method without the necessary characteristics are less obvious but no less important. In a number of patient groups, including pregnant women, intensive care patients, patients with liver failure, haemodialysis patients and osteoporotic patients, automated immunoassays have proven unreliable. Measuring 25(OH)D in these patients with an automated immunoassay is therefore not advised. As there is usually no prior knowledge of the possible interferences in a patient sample, LC-MS/MS is the preferred method for 25(OH)D determination in patient diagnostics.

All in all, measuring vitamin D metabolism has greatly benefitted from the entry of LC-MS/MS in clinical laboratories. It enabled us to drastically improve specificity and accuracy, and provided us with the ability to co-measure multiple metabolites simultaneously. It has already proven very useful in the diagnosis of numerous conditions and new generations of more sensitive LC-MS/MS instruments will enable us to further meticulously study, to date unexplained, vitamin D metabolism-related conditions. If immunoassay manufacturers succeed in improving their newer generations of automated immunoassays, as has happened with the 2nd generation testosterone immunoassays, they may overcome the problems they face today and prove reliable in patient diagnostics [98]. By the same token, the disadvantages of today’s LC-MS/MS instruments, being quite laborious, may be resolved by more easy to use instruments and the introduction of fully automated LC-MS/MS systems. Additionally, further efforts in the standardisation of assays are needed to raise the overall quality of these methods and provide clinicians with reliable results.

Conclusively, measuring vitamin D metabolites is, and will be important, to diagnose a number of different conditions. Attention to the when, the what and the how of measuring vitamin D metabolism greatly benefits diagnostic and research power.

## Figures and Tables

**Table 1:** Advantages and disadvantages of current LC-MS/MS methods and immunoassays for vitamin D metabolism determination.

Metabolite	LC-MS/MS		Immunoassay	
25(OH)D	Advantages: Sample preparation adaptable Specificity	Disadvantages: Complexity Difficult to separate epi-25(OH)D_3_ from 25(OH)D_3_ (same mass and chromatographic behaviour)	Advantages *: Fast Easy operation No cross reactivity with epi-25(OH)D	Disadvantages: Patient group-dependent deviations No distinction between 25(OH)D_2_ and 25(OH)D_3_ Cross-reactivity of other vitamin D metabolites recognised by the antibody, such as 24,25(OH)_2_D
1,25(OH)_2_D	Advantages: Sample preparation adaptable Specificity	Disadvantages: Complexity Sensitivity Possible cross-reactivity from isobaric interferences	Advantages: Fast Easy operation Sensitivity	Disadvantages: No distinction between 1,25(OH)_2_D_2_ and 1,25(OH)_2_D_3_ Cross-reactivity of other vitamin D metabolites recognised by the antibody, such as 25(OH)D_3_ and 24,25(OH)_2_D
24,25(OH)_2_D	Advantages: Sample preparation adaptable Specificity	Disadvantages: Complexity	N/A ^†^	N/A ^†^

* The advantages listed are limited to the automated immunoassays. ^†^ Although 24,25(OH)_2_D immunoassays exist, too little information on their performance is available.

## References

[1] Heaney R.P., Recker R.R., Grote J., Horst R.L., Armas L.A. (2011). Vitamin D(3) is more potent than vitamin D(2) in humans. J. Clin. Endocrinol. Metab..

[2] Kamao M., Tatematsu S., Hatakeyama S., Sakaki T., Sawada N., Inouye K., Ozono K., Kubodera N., Reddy G.S., Okano T. (2004). C-3 epimerization of vitamin D_3_ metabolites and further metabolism of C-3 epimers: 25-hydroxyvitamin D_3_ is metabolized to 3-epi-25-hydroxyvitamin D_3_ and subsequently metabolized through C-1alpha or C-24 hydroxylation. J. Biol. Chem..

[3] Saito H., Kusano K., Kinosaki M., Ito H., Hirata M., Segawa H., Miyamoto K., Fukushima N. (2003). Human fibroblast growth factor-23 mutants suppress Na^+^-dependent phosphate co-transport activity and 1alpha,25-dihydroxyvitamin D_3_ production. J. Biol. Chem..

[4] Bikle D. (2009). Nonclassic actions of vitamin D. J. Clin. Endocrinol. Metab..

[5] Liu P.T., Stenger S., Li H., Wenzel L., Tan B.H., Krutzik S.R., Ochoa M.T., Schauber J., Wu K., Meinken C. (2006). Toll-like receptor triggering of a vitamin D-mediated human antimicrobial response. Science.

[6] Lips P. (2007). Relative value of 25(OH)D and 1,25(OH)_2_D measurements. J. Bone Miner. Res. Off. J. Am. Soc. Bone Miner. Res..

[7] Holick M.F. (2007). Vitamin D deficiency. N. Engl. J. Med..

[8] Holick M.F. (2009). Vitamin D status: Measurement, interpretation, and clinical application. Ann. Epidemiol..

[9] Hollis B.W. (2010). Assessment and interpretation of circulating 25-hydroxyvitamin D and 1,25-dihydroxyvitamin D in the clinical environment. Endocrinol. Metab. Clin. North Am..

[10] Bouillon R., Van Schoor N.M., Gielen E., Boonen S., Mathieu C., Vanderschueren D., Lips P. (2013). Optimal vitamin D status: A critical analysis on the basis of evidence-based medicine. J. Clin. Endocrinol. Metab..

[11] Ross A.C., Manson J.E., Abrams S.A., Aloia J.F., Brannon P.M., Clinton S.K., Durazo-Arvizu R.A., Gallagher J.C., Gallo R.L., Jones G. (2011). The 2011 report on dietary reference intakes for calcium and vitamin D from the institute of medicine: What clinicians need to know. J. Clin. Endocrinol. Metab..

[12] Manson J.E., Brannon P.M., Rosen C.J., Taylor C.L. (2016). Vitamin D deficiency—Is there really a pandemic?. N. Engl. J. Med..

[13] Bischoff-Ferrari H.A., Giovannucci E., Willett W.C., Dietrich T., Dawson-Hughes B. (2006). Estimation of optimal serum concentrations of 25-hydroxyvitamin d for multiple health outcomes. Am. J. Clin. Nutr..

[14] Norman A.W., Bouillon R., Whiting S.J., Vieth R., Lips P. (2007). 13th workshop consensus for vitamin D nutritional guidelines. J. Steroid Biochem. Mol. Biol..

[15] Ross A.C. (2011). The 2011 report on dietary reference intakes for calcium and vitamin D. Public Health Nutr..

[16] Holick M.F., Binkley N.C., Bischoff-Ferrari H.A., Gordon C.M., Hanley D.A., Heaney R.P., Murad M.H., Weaver C.M. (2011). Evaluation, treatment, and prevention of vitamin D deficiency: An endocrine society clinical practice guideline. J. Clin. Endocrinol. Metab..

[17] Dirks N.F., Martens F., Vanderschueren D., Billen J., Pauwels S., Ackermans M.T., Endert E., Heijer M.D., Blankenstein M.A., Heijboer A.C. (2016). Determination of human reference values for serum total 1,25-dihydroxyvitamin D using an extensively validated 2D ID-UPLC-MS/MS method. J. Steroid Biochem. Mol. Biol..

[18] Zalewski A., Ma N.S., Legeza B., Renthal N., Fluck C.E., Pandey A.V. (2016). Vitamin D-dependent rickets type 1 caused by mutations in CYP27B1 affecting protein interactions with adrenodoxin. J. Clin. Endocrinol. Metab..

[19] Francis F., Hennig S., Korn B., Reinhardrdt R., de Jong P., Poustka A., Lehrach H., Rowe P.S.N., Goulding J.N., Summerfield T. (1995). A gene (PEX) with homologies to endopeptidases is mutated in patients with x-linked hypophosphatemic rickets. The HYP consortium. Nat. Genet..

[20] Imel E.A., DiMeglio L.A., Hui S.L., Carpenter T.O., Econs M.J. (2010). Treatment of x-linked hypophosphatemia with calcitriol and phosphate increases circulating fibroblast growth factor 23 concentrations. J. Clin. Endocrinol. Metab..

[21] Chong W.H., Molinolo A.A., Chen C.C., Collins M.T. (2011). Tumor-induced osteomalacia. Endocr.-Relat. Cancer.

[22] Nagata Y., Imanishi Y., Ishii A., Kurajoh M., Motoyama K., Morioka T., Naka H., Mori K., Miki T., Emoto M. (2011). Evaluation of bone markers in hypophosphatemic rickets/osteomalacia. Endocrine.

[23] Consortium A. (2000). Autosomal dominant hypophosphataemic rickets is associated with mutations in FGF23. Nat. Genet..

[24] White K.E., Carn G., Lorenz-Depiereux B., Benet-Pages A., Strom T.M., Econs M.J. (2001). Autosomal-dominant hypophosphatemic rickets (ADHR) mutations stabilize FGF-23. Kidney Int..

[25] Levy-Litan V., Hershkovitz E., Avizov L., Leventhal N., Bercovich D., Chalifa-Caspi V., Manor E., Buriakovsky S., Hadad Y., Goding J. (2010). Autosomal-recessive hypophosphatemic rickets is associated with an inactivation mutation in the ENPP1 gene. Am. J. Hum. Genet..

[26] Rafaelsen S.H., Raeder H., Fagerheim A.K., Knappskog P., Carpenter T.O., Johansson S., Bjerknes R. (2013). Exome sequencing reveals FAM20c mutations associated with fibroblast growth factor 23-related hypophosphatemia, dental anomalies, and ectopic calcification. J. Bone Miner. Res..

[27] Riminucci M., Collins M.T., Fedarko N.S., Cherman N., Corsi A., White K.E., Waguespack S., Gupta A., Hannon T., Econs M.J. (2003). FGF-23 in fibrous dysplasia of bone and its relationship to renal phosphate wasting. J. Clin. Investig..

[28] Farrow E.G., Davis S.I., Mooney S.D., Beighton P., Mascarenhas L., Gutierrez Y.R., Pitukcheewanont P., White K.E. (2006). Extended mutational analyses of FGFR1 in osteoglophonic dysplasia. Am. J. Med. Genet. Part A.

[29] Avitan-Hersh E., Tatur S., Indelman M., Gepstein V., Shreter R., Hershkovitz D., Brick R., Bergman R., Tiosano D. (2014). Postzygotic HRAS mutation causing both keratinocytic epidermal nevus and thymoma and associated with bone dysplasia and hypophosphatemia due to elevated FGF23. J. Clin. Endocrinol. Metab..

[30] Gupta A., Dwivedi A., Patel P., Gupta S. (2015). Hypophosphatemic osteomalacia in von recklinghausen neurofibromatosis: Case report and literature review. Indian J. Radiol. Imaging.

[31] Brownstein C.A., Adler F., Nelson-Williams C., Iijima J., Li P., Imura A., Nabeshima Y., Reyes-Mugica M., Carpenter T.O., Lifton R.P. (2008). A translocation causing increased alpha-klotho level results in hypophosphatemic rickets and hyperparathyroidism. Proc. Natl. Acad. Sci. USA.

[32] Brown W.W., Juppner H., Langman C.B., Price H., Farrow E.G., White K.E., McCormick K.L. (2009). Hypophosphatemia with elevations in serum fibroblast growth factor 23 in a child with jansen’s metaphyseal chondrodysplasia. J. Clin. Endocrinol. Metab..

[33] Chanakul A., Zhang M.Y., Louw A., Armbrecht H.J., Miller W.L., Portale A.A., Perwad F. (2013). FGF-23 regulates CYP27B1 transcription in the kidney and in extra-renal tissues. PLoS ONE.

[34] Lorenz-Depiereux B., Benet-Pages A., Eckstein G., Tenenbaum-Rakover Y., Wagenstaller J., Tiosano D., Gershoni-Baruch R., Albers N., Lichtner P., Schnabel D. (2006). Hereditary hypophosphatemic rickets with hypercalciuria is caused by mutations in the sodium-phosphate cotransporter gene SLC34A3. Am. J. Hum. Genet..

[35] Tieder M., Modai D., Shaked U., Samuel R., Arie R., Halabe A., Maor J., Weissgarten J., Averbukh Z., Cohen N. (1987). “Idiopathic” hypercalciuria and hereditary hypophosphatemic rickets. Two phenotypical expressions of a common genetic defect. N. Engl. J. Med..

[36] Goto S., Fujii H., Kono K., Watanabe K., Nakai K., Nishi S. (2016). Serum FGF23 levels may not be associated with serum phosphate and 1,25-dihydroxyvitamin D levels in patients with fanconi syndrome–induced hypophosphatemia. Clin. Kidney J..

[37] Adams J.S., Hewison M. (2012). Extrarenal expression of the 25-hydroxyvitamin d-1-hydroxylase. Arch. Biochem. Biophys..

[38] Donovan P.J., Sundac L., Pretorius C.J., d’Emden M.C., McLeod D.S. (2013). Calcitriol-mediated hypercalcemia: Causes and course in 101 patients. J. Clin. Endocrinol. Metab..

[39] Abreu M.T., Kantorovich V., Vasiliauskas E.A., Gruntmanis U., Matuk R., Daigle K., Chen S., Zehnder D., Lin Y.C., Yang H. (2004). Measurement of vitamin D levels in inflammatory bowel disease patients reveals a subset of crohn’s disease patients with elevated 1,25-dihydroxyvitamin D and low bone mineral density. Gut.

[40] Karakelides H., Geller J.L., Schroeter A.L., Chen H., Behn P.S., Adams J.S., Hewison M., Wermers R.A. (2006). Vitamin D-mediated hypercalcemia in slack skin disease: Evidence for involvement of extrarenal 25-hydroxyvitamin D 1alpha-hydroxylase. J. Bone Miner. Res. Off. J. Am. Soc. Bone Miner. Res..

[41] Jones G., Strugnell S.A., DeLuca H.F. (1998). Current understanding of the molecular actions of vitamin D. Physiol. Rev..

[42] St-Arnaud R., Glorieux F.H. (1998). 24,25-dihydroxyvitamin D—Active metabolite or inactive catabolite?. Endocrinology.

[43] St-Arnaud R. (1999). Novel findings about 24,25-dihydroxyvitamin D: An active metabolite?. Curr. Opin. Nephrol. Hypertens..

[44] Wagner D., Hanwell H.E., Schnabl K., Yazdanpanah M., Kimball S., Fu L., Sidhom G., Rousseau D., Cole D.E., Vieth R. (2011). The ratio of serum 24,25-dihydroxyvitamin D(3) to 25-hydroxyvitamin D(3) is predictive of 25-hydroxyvitamin D(3) response to vitamin D(3) supplementation. J. Steroid Biochem. Mol. Biol..

[45] Kaufmann M., Gallagher J.C., Peacock M., Schlingmann K.P., Konrad M., DeLuca H.F., Sigueiro R., Lopez B., Mourino A., Maestro M. (2014). Clinical utility of simultaneous quantitation of 25-hydroxyvitamin D and 24,25-dihydroxyvitamin D by LC-MS/MS involving derivatization with DMEQ-TAD. J. Clin. Endocrinol. Metab..

[46] Schlingmann K.P., Kaufmann M., Weber S., Irwin A., Goos C., John U., Misselwitz J., Klaus G., Kuwertz-Broking E., Fehrenbach H. (2011). Mutations in CYP24A1 and idiopathic infantile hypercalcemia. N. Engl. J. Med..

[47] Dinour D., Beckerman P., Ganon L., Tordjman K., Eisenstein Z., Holtzman E.J. (2013). Loss-of-function mutations of CYP24A1, the vitamin D 24-hydroxylase gene, cause long-standing hypercalciuric nephrolithiasis and nephrocalcinosis. J. Urol..

[48] Sayers J., Hynes A.M., Srivastava S., Dowen F., Quinton R., Datta H.K., Sayer J.A. (2015). Successful treatment of hypercalcaemia associated with a CYP24A1 mutation with fluconazole. Clin. Kidney J..

[49] Nesterova G., Malicdan M.C., Yasuda K., Sakaki T., Vilboux T., Ciccone C., Horst R., Huang Y., Golas G., Introne W. (2013). 1,25-(OH)_2_D-24 hydroxylase (CYP24A1) deficiency as a cause of nephrolithiasis. Clin. J. Am. Soc. Nephrol. CJASN.

[50] Molin A., Baudoin R., Kaufmann M., Souberbielle J.C., Ryckewaert A., Vantyghem M.C., Eckart P., Bacchetta J., Deschenes G., Kesler-Roussey G. (2015). CYP24A1 mutations in a cohort of hypercalcemic patients: Evidence for a recessive trait. J. Clin. Endocrinol. Metab..

[51] Cashman K.D., Hayes A., Galvin K., Merkel J., Jones G., Kaufmann M., Hoofnagle A.N., Carter G.D., Durazo-Arvizu R.A., Sempos C.T. (2015). Significance of serum 24,25-dihydroxyvitamin D in the assessment of vitamin D status: A double-edged sword?. Clin. Chem..

[52] Depreter B., Heijboer A.C., Langlois M.R. (2013). Accuracy of three automated 25-hydroxyvitamin D assays in hemodialysis patients. Clin. Chim. Acta Int. J. Clin. Chem..

[53] Ong L., Saw S., Sahabdeen N.B., Tey K.T., Ho C.S., Sethi S.K. (2012). Current 25-hydroxyvitamin D assays: Do they pass the test?. Clin. Chim. Acta Int. J. Clin. Chem..

[54] Farrell C.J., Martin S., McWhinney B., Straub I., Williams P., Herrmann M. (2012). State-of-the-art vitamin D assays: A comparison of automated immunoassays with liquid chromatography-tandem mass spectrometry methods. Clin. Chem..

[55] Heijboer A.C., Blankenstein M.A., Kema I.P., Buijs M.M. (2012). Accuracy of 6 routine 25-hydroxyvitamin D assays: Influence of vitamin d binding protein concentration. Clin. Chem..

[56] Wise S.A., Phinney K.W., Tai S.S., Camara J.E., Myers G.L., Durazo-Arvizu R., Tian L., Hoofnagle A.N., Bachmann L.M., Young I.S. (2017). Baseline assessment of 25-hydroxyvitamin D assay performance: A vitamin D standardization program (VDSP) interlaboratory comparison study. J. AOAC Int..

[57] Cavalier E., Lukas P., Bekaert A.C., Peeters S., Le Goff C., Yayo E., Delanaye P., Souberbielle J.C. (2016). Analytical and clinical evaluation of the new Fujirebio Lumipulse® G non-competitive assay for 25(OH)-vitamin D and three immunoassays for 25(OH)D in healthy subjects, osteoporotic patients, third trimester pregnant women, healthy African subjects, hemodialyzed and intensive care patients. Clin. Chem. Lab. Med..

[58] Elsenberg E., Ten Boekel E., Huijgen H., Heijboer A.C. (2017). Standardization of automated 25-hydroxyvitamin D assays: How successful is it?. Clin. Biochem..

[59] Freeman J., Wilson K., Spears R., Shalhoub V., Sibley P. (2014). Influence of vitamin d binding protein on accuracy of 25-hydroxyvitamin D measurement using the advia centaur vitamin d total assay. Int. J. Endocrinol..

[60] Hsu S.A., Soldo J., Gupta M. (2013). Evaluation of two automated immunoassays for 25-OH vitamin D: Comparison against LC-MS/MS. J. Steroid Biochem. Mol. Biol..

[61] Janssen M.J., Wielders J.P., Bekker C.C., Boesten L.S., Buijs M.M., Heijboer A.C., van der Horst F.A., Loupatty F.J., van den Ouweland J.M. (2012). Multicenter comparison study of current methods to measure 25-hydroxyvitamin D in serum. Steroids.

[62] Chouiali A., Mallet P.L., Fink G., Biron S., Langlois M.F. (2017). Comparison of two methods for measuring 25-OH vitamin D in the follow-up of patients after bilio-pancreatic diversion bariatric surgery. Clin. Biochem..

[63] Brock A.T., Strickland S.W., Bazydlo L.A.L., Haverstick D.M. (2017). An underestimation of 25-OH vitamin D in patients with renal disease by the abbott architect immunoassay. J. Appl. Lab. Med. AACC Publ..

[64] Shu I., Pina-Oviedo S., Quiroga-Garza G., Meng Q.H., Wang P. (2013). Influence of vitamin D_2_ percentage on accuracy of 4 commercial total 25-hydroxyvitamin D assays. Clin. Chem..

[65] Tolan N.V., Yoon E.J., Brady A.R., Horowitz G.L. (2017). Price of high-throughput 25-hydroxyvitamin D immunoassays: Frequency of inaccurate results. J. Appl. Lab. Med. AACC Publ..

[66] Le Goff C., Peeters S., Crine Y., Lukas P., Souberbielle J.C., Cavalier E. (2012). Evaluation of the cross-reactivity of 25-hydroxyvitamin D_2_ on seven commercial immunoassays on native samples. Clin. Chem. Lab. Med..

[67] Carter G.D., Jones J.C., Shannon J., Williams E.L., Jones G., Kaufmann M., Sempos C. (2016). 25-hydroxyvitamin D assays: Potential interference from other circulating vitamin D metabolites. J. Steroid Biochem. Mol. Biol..

[68] Dowling K.G., Hull G., Sundvall J., Lamberg-Allardt C., Cashman K.D. (2017). Improved accuracy of an tandem liquid chromatography-mass spectrometry method measuring 24R,25-dihydroxyvitamin D_3_ and 25-hydroxyvitamin D metabolites in serum using unspiked controls and its application to determining cross-reactivity of a chemiluminescent microparticle immunoassay. J. Chromatogr. A.

[69] Burdette C.Q., Camara J.E., Nalin F., Pritchett J., Sander L.C., Carter G.D., Jones J., Betz J.M., Sempos C.T., Wise S.A. (2017). Establishing an accuracy basis for the vitamin D external quality assessment scheme (DEQAS). J. AOAC Int..

[70] Ooms N., van Daal H., Beijers A.M., Gerrits G.P., Semmekrot B.A., van den Ouweland J.M. (2016). Time-course analysis of 3-epi-25-hydroxyvitamin D_3_ shows markedly elevated levels in early life, particularly from vitamin D supplementation in preterm infants. Pediatr. Res..

[71] Van den Ouweland J.M., Beijers A.M., van Daal H. (2014). Overestimation of 25-hydroxyvitamin D_3_ by increased ionisation efficiency of 3-epi-25-hydroxyvitamin D_3_ in LC-MS/MS methods not separating both metabolites as determined by an LC-MS/MS method for separate quantification of 25-hydroxyvitamin D_3_, 3-epi-25-hydroxyvitamin D_3_ and 25-hydroxyvitamin D_2_ in human serum. J. Chromatogr. B Anal. Technol. Biomed. Life Sci..

[72] Carter G.D. (2011). Accuracy of 25-hydroxyvitamin D assays: Confronting the issues. Curr. Drug Targets.

[73] Fabregat-Cabello N., Farre-Segura J., Huyghebaert L., Peeters S., Le Goff C., Souberbielle J.-C., Cavalier É. (2017). A fast and simple method for simultaneous measurements of 25(OH)D, 24,25(OH)_2_D and the vitamin D metabolite ratio (VMR) in serum samples by LC-MS/MS. Clin. Chim. Acta.

[74] Jenkinson C., Taylor A.E., Hassan-Smith Z.K., Adams J.S., Stewart P.M., Hewison M., Keevil B.G. (2016). High throughput LC-MS/MS method for the simultaneous analysis of multiple vitamin D analytes in serum. J. Chromatogr. B Anal. Technol. Biomed. Life Sci..

[75] Muller M.J., Stokes C.S., Lammert F., Volmer D.A. (2016). Chemotyping the distribution of vitamin D metabolites in human serum. Sci. Rep..

[76] Van den Ouweland J.M., Beijers A.M., van Daal H. (2011). Fast separation of 25-hydroxyvitamin D_3_ from 3-epi-25-hydroxyvitamin D_3_ in human serum by liquid chromatography-tandem mass spectrometry: Variable prevalence of 3-epi-25-hydroxyvitamin D_3_ in infants, children, and adults. Clin. Chem..

[77] Carter G.D., Jones J.C. (2009). Use of a common standard improves the performance of liquid chromatography-tandem mass spectrometry methods for serum 25-hydroxyvitamin-D. Ann. Clin. Biochem..

[78] Dirks N.F., Vesper H.W., van Herwaarden A.E., van den Ouweland J.M., Kema I.P., Krabbe J.G., Heijboer A.C. (2016). Various calibration procedures result in optimal standardization of routinely used 25(OH)D ID-LC-MS/MS methods. Clin. Chim. Acta Int. J. Clin. Chem..

[79] Hawkes C.P., Schnellbacher S., Singh R.J., Levine M.A. (2015). 25-hydroxyvitamin D can interfere with a common assay for 1,25-dihydroxyvitamin D in vitamin D intoxication. J. Clin. Endocrinol. Metab..

[80] Strathmann F.G., Laha T.J., Hoofnagle A.N. (2011). Quantification of 1alpha,25-dihydroxy vitamin D by immunoextraction and liquid chromatography-tandem mass spectrometry. Clin. Chem..

[81] Kimball S.M., Vieth R. (2007). A comparison of automated methods for the quantitation of serum 25-hydroxyvitamin D and 1,25-dihydroxyvitamin D. Clin. Biochem..

[82] Zittermann A., Ernst J.B., Becker T., Dreier J., Knabbe C., Gummert J.F., Kuhn J. (2016). Measurement of circulating 1,25-dihydroxyvitamin D: Comparison of an automated method with a liquid chromatography tandem mass spectrometry method. Int. J. Anal. Chem..

[83] Valcour A., Zierold C., Podgorski A.L., Olson G.T., Wall J.V., DeLuca H.F., Bonelli F. (2016). A novel, fully-automated, chemiluminescent assay for the detection of 1,25-dihydroxyvitamin D in biological samples. J. Steroid Biochem. Mol. Biol..

[84] Miller N., Gruson D. (2016). Implementation of automated testing for 1,25-dihydroxyvitamin D: Return of experience from a core-laboratory. Clin. Biochem..

[85] Pauwels S., Jans I., Billen J., Heijboer A., Verstuyf A., Carmeliet G., Mathieu C., Maestro M., Waelkens E., Evenepoel P. (2017). 1beta,25-dihydroxyvitamin D_3_: A new vitamin d metabolite in human serum. J. Steroid Biochem. Mol. Biol..

[86] Duan X., Weinstock-Guttman B., Wang H., Bang E., Li J., Ramanathan M., Qu J. (2010). Ultrasensitive quantification of serum vitamin D metabolites using selective solid-phase extraction coupled to microflow liquid chromatography and isotope-dilution mass spectrometry. Anal. Chem..

[87] Hedman C.J., Wiebe D.A., Dey S., Plath J., Kemnitz J.W., Ziegler T.E. (2014). Development of a sensitive LC/MS/MS method for vitamin D metabolites: 1,25 dihydroxyvitamin D_2&3_ measurement using a novel derivatization agent. J. Chromatogr. B Anal. Technol. Biomed. Life Sci..

[88] Wang Z., Senn T., Kalhorn T., Zheng X.E., Zheng S., Davis C.L., Hebert M.F., Lin Y.S., Thummel K.E. (2011). Simultaneous measurement of plasma vitamin D(3) metabolites, including 4beta,25-dihydroxyvitamin D(3), using liquid chromatography-tandem mass spectrometry. Anal. Biochem..

[89] Casetta B., Jans I., Billen J., Vanderschueren D., Bouillon R. (2010). Development of a method for the quantification of 1alpha,25(OH)_2_-vitamin D_3_ in serum by liquid chromatography tandem mass spectrometry without derivatization. Eur. J. Mass Spectrom..

[90] Fang H., Yu S., Cheng Q., Cheng X., Han J., Qin X., Xia L., Jiang X., Qiu L. (2016). Determination of 1,25-dihydroxyvitamin D_2_ and 1,25-dihydroxyvitamin D_3_ in human serum using liquid chromatography with tandem mass spectrometry. J. Chromatogr. B Anal. Technol. Biomed. Life Sci..

[91] Kissmeyer A.M., Sonne K. (2001). Sensitive analysis of 1alpha,25-dihydroxyvitamin D_3_ in biological fluids by liquid chromatography-tandem mass spectrometry. J. Chromatogr. A.

[92] Yuan C., Kosewick J., He X., Kozak M., Wang S. (2011). Sensitive measurement of serum 1alpha,25-dihydroxyvitamin D by liquid chromatography/tandem mass spectrometry after removing interference with immunoaffinity extraction. Rapid Commun. Mass Spectrom..

[93] Tang J.C.Y., Nicholls H., Piec I., Washbourne C.J., Dutton J.J., Jackson S., Greeves J., Fraser W.D. (2017). Reference intervals for serum 24,25-dihydroxyvitamin D and the ratio with 25-hydroxyvitamin D established using a newly developed LC–MS/MS method. J. Nutr. Biochem..

[94] Baecher S., Leinenbach A., Wright J.A., Pongratz S., Kobold U., Thiele R. (2012). Simultaneous quantification of four vitamin D metabolites in human serum using high performance liquid chromatography tandem mass spectrometry for vitamin D profiling. Clin. Biochem..

[95] Mena-Bravo A., Priego-Capote F., Luque de Castro M.D. (2015). Study of blood collection and sample preparation for analysis of vitamin D and its metabolites by liquid chromatography-tandem mass spectrometry. Anal. Chim. Acta.

[96] Tai S.S., Nelson M.A. (2015). Candidate reference measurement procedure for the determination of (24R),25-dihydroxyvitamin D_3_ in human serum using isotope-dilution liquid chromatography-tandem mass spectrometry. Anal. Chem..

[97] Wise S.A., Tai S.S., Nelson M.A., Burdette C.Q., Camara J.E., Hoofnagle A.N., Laha T.J., Carter G.D., Jones J., Williams E.L. (2017). Interlaboratory comparison for the determination of 24,25-dihydroxyvitamin D(3) in human serum using liquid chromatography with tandem mass spectrometry. J. AOAC Int..

[98] Groenestege W.M.T., Bui H.N., Kate J.T., Menheere P.P.C.A., Oosterhuis W.P., Vader H.L., Heijboer A.C., Janssen M.J.W. (2012). Accuracy of first and second generation testosterone assays and improvement through sample extraction. Clin. Chem..

